# The clockwork of insect activity: Advancing ecological understanding through automation

**DOI:** 10.1111/1365-2656.14246

**Published:** 2025-01-24

**Authors:** Ameli Kirse, Manus Arian Wittenhorst, Christoph Scherber, Michel Posanski, Alice Scherges, Vera Zizka, David Ott, Niklas W. Noll, Wolfgang J. Wägele

**Affiliations:** ^1^ Leibniz Institute for the Analysis of Biodiversity Change (LIB), Museum Koenig, Centre for Biodiversity Monitoring and Conservation Science Bonn Germany; ^2^ Bonn Institute for Organismic Biology (BIOB) University of Bonn Bonn Germany

**Keywords:** activity patterns, automated monitoring, biodiversity monitoring, chronoecology, circadian rhythm, insects, metabarcoding, technology

## Abstract

1. Understanding insect behaviour and its underlying drivers is vital for interpreting changes in local biodiversity and predicting future trends. Conventional insect traps are typically limited to assess the composition of local insect communities over longer time periods and provide only limited insights into the effects of abiotic factors, such as light on species activity. Achieving finer temporal resolution is labour‐intensive or only possible under laboratory conditions.
2. Here, we demonstrate that time‐controlled insect sampling using an automated Malaise trap in combination with metabarcoding allows for the observation and documentation of taxon‐specific activity patterns. Furthermore, these recorded activity patterns can provide valuable insights into the underlying ecological processes.
3. Insect activity curves, derived from predicted detection numbers using generalised linear latent variable models, reveal distinct differences in activity patterns at higher and lower taxonomic level. While our findings align with existing literature, they also reveal that the activity patterns of some species are more complex than previously known. Additionally, a comparison of the assessed activity patterns across taxa suggest potential, previously undescribed parasitoid–host relationships. Within taxonomic groups, we observe variations in both the timing and duration of activity patterns, which can be linked to differences in mating strategies among closely related species.
4. By capturing circadian rhythms of insect activity through time‐controlled bulk sampling, we can expand our knowledge on species behaviour, ecology and temporal interactions. This contributes significantly to the advancement of chronoecology, allowing for further exploration of the roles of species and benefits in natural and anthropogenic ecosystems, alongside their potentially significant threat.

## Graphical abstract

The study demonstrates that time‐controlled insect sampling using an automated Malaise trap in combination with metabarcoding allows for the observation and documentation of taxon‐specific activity patterns. These recorded activity patterns can provide valuable insights into the underlying ecological processes, including parasitoid–host relationships and mating strategies.

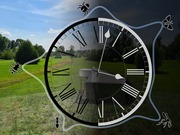

## 1 INTRODUCTION

In an era marked by climate change and biodiversity loss, it is crucial to enhance our understanding of insect activity patterns and the underlying causes, triggers and catalysts behind them. Such comprehension enables us to rigorously interpret monitored shifts in diversity patterns within local communities and will facilitate the development of predictive models to assess future trends. Nature follows a temporal organisation regulated by the rhythms of seasons, day and night, lunar phases and tides. Most organisms rely on internal biological clocks to predict and prepare for these recurring alterations (Bass, 2012). Temporal synchronisation within biological systems is encoded in the genetic framework, enabling cells to discern and respond to temporal cues and thus, facilitating the orchestration of an organism's physiological and biochemical functions in rhythmic patterns aligned with daily, monthly or yearly environmental changes (Kreitzman & Foster, 2011). Critical physiological processes, encompassing sleep and hormone production, exhibit time‐dependent variations, intricately linked to the diurnal cycle determined by the Sun's position and the ensuing alternation of day and night. Furthermore, specific biological behaviours, such as mating, migration, hibernation and flowering, follow cyclic patterns responding to predictable environmental cues, including variations in temperature, humidity and food availability (Kreitzman & Foster, 2011).

Chronobiology, the study of biological rhythms, deals with the functioning of these internal clocks (Dunlap et al., 2004), while the newly emerging field of chronoecology explores the effects on the survival of the species (Halle & Stenseth, 2000; Muñoz‐Delgado & Corsi‐Cabrera, 2010).

Biological clocks can inter alia control seasonal (e.g. bird migration and hibernation) (Åkesson et al., 2017; Körtner & Geiser, 2000), but also daily rhythms (e.g. activity patterns) of physiology and behaviour (Patke et al., 2020). The vast majority of organisms have evolved mechanisms to predict and align with the recurring environmental circadian cycles that span approximately 24 h (Begemann et al., 2020; Pilorz et al., 2018). The daily cycle of night and day, referred to as ‘diel’, serves to synchronise activity patterns. There are three main diel activity patterns: ‘diurnal’ (occurring during the day), ‘nocturnal’ (active during the night), and ‘crepuscular’ (active during dawn and dusk) (Binder et al., 2011). Next to abiotic stressors, biotic factors can also be the driving force for observed diel activities, for example predator avoidance (van der Veen et al., 2017). Advancements in understanding the dynamics of insect movement have been driven by recent technological innovations. In this context, the utilisation of remote light detection and ranging (lidar) systems has shown effectiveness in measuring insect movement and corresponding activity patterns (Kirkeby et al., 2016). However, it is important to note that these systems can only differentiate between insects on a higher taxonomic level and do not facilitate the distinction between species. Thus, comprehensive data regarding the typical daily activity patterns are missing for the majority of insect species, as continuous monitoring has long been only possible under laboratory conditions (Dominoni et al., 2017; Kronfeld‐Schor et al., 2013).

Commonly used trap systems (e.g. Malaise traps, Vane traps and Pitfalls traps) are effective tools for monitoring insects (Geiger et al., 2016). However, identifying multi‐taxon insect samples based on morphological traits requires group‐specific skilled experts, making the process costly and often time‐consuming. As a result, even when sampling is performed with a generalistic insect trap, studies mainly concentrate on a limited set of taxa (Strumia, 2011) or bypass time‐consuming species identification by utilising biomass as a proxy for insect diversity and activity (Hallmann et al., 2017), which excludes valuable information. Today, metabarcoding allows the timely taxonomic identification of several thousands of specimens in parallel (Kirse et al., 2023; Sickel et al., 2023). Metabarcoding combines the concept of barcoding with high‐throughput sequencing. To cover a broad spectrum of insects, universal primers are used, which however do not work equally well for all arthropod groups. For example, it can be challenging to assess certain hymenopterans groups, due to inconsistencies in the degree of conservation in the primer binding region (Brandon‐Mong et al., 2015; Elbrecht et al., 2019; Kirse et al., 2021). Additionally, reference databases are still expanding. In Germany, the German Barcode of Life initiative was launched in 2011 and is still generating barcodes for many species of Germany (Caruso et al., 2024). As the power of metabarcoding is directly linked to the completeness of reference databases, this implies that species that have not yet been barcoded cannot be identified through metabarcoding, leading to false negative results.

Although species identification can now be conducted in a comparatively short time, the collection of specimens still requires a high amount of human resources, especially when aiming to assess continuous data of diel insect activity patterns (Zoller et al., 2020). Modern tools, designed for insect monitoring based on computer vision and sound recognition, are promising to circumvent those difficulties as collected sound and image data usually come with a timestamp. However, development and establishment of reference databases are still in an early stage, lacking references for the majority of insect species (van Klink et al., 2022). Additionally, most of the new systems are only trained to identify a certain set of species, whereas especially with sound recognition, multiple recordings of the same individual could distort the results (Kułaga & Budka, 2019).

Automating conventional insect traps addresses the limitations described above. As early as 1965, Lewis and Taylor employed an automated trap system, specifically a 9‐inch Ventaxia or 12‐inch Aerofoil suction trap, for insect collection. These traps were advanced for their time, capable of subdividing collected specimens into up to 48 separate samples over a 24‐h cycle (Taylor, 1962). However, they had several limitations, including reliance on an external power source, restrictions on capturing larger insects, the need for routine maintenance and daily sample collection, and the risk of cross‐contamination between samples due to the rudimentary in‐built sample separation mechanism.

In 2011, Strumia enhanced this concept by combining an automated sampling device with a Malaise trap, allowing for the simultaneous assessment of circadian activity patterns in certain flying insect groups. The Strumia trap accommodates eight collection bottles and can operate autonomously in the field for up to 3 weeks (Strumia, 2011). Following a similar approach, we combined a Townes Malaise trap with an automated sampling system called the ‘AMMOD multisampler’ (name derived from the AMMOD project—Automated Multisensor Stations for BioDiversity) (Kirse et al., 2024). The AMMOD multisampler operates on solar power, enabling continuous monitoring over several months without human intervention, even in remote areas where an external power source may not be available. Similar to the Strumia trap, the AMMOD multisampler can change collection bottles in predetermined time intervals, facilitating time‐controlled and continuous assessment of flying insect activity throughout both day and night. However, with the capacity to hold 12 collection bottles compared with the 8‐collection‐bottle capacity of the Strumia trap, the AMMOD multisampler allows for a more fine‐grained documentation of insect activity. For our study, each bottle was placed under the catching device 14 times, resulting in 12 bulk samples, each combining insect catches collected in a pre‐defined time period of 2 h on 14 consecutive days. The collected samples were stored in an individual 1000‐mL bottle containing a preservative fluid, ensuring optimal preservation of genetic material for subsequent species identification using molecular tools. Here, we have utilised a metabarcoding protocol for high‐resolution taxonomic identification of bulk samples to increase the taxonomic range and resolution and thus information output. It is yet not possible to provide reliable and exact abundance data through DNA metabarcoding, but semi‐quantitative metrics like relative read abundance (RRA) can be used as an informative metric for approximate abundances in a system (Sickel et al., 2023). However, species presence–absence lists can already provide valuable information about the status of the associated ecosystem. By combining an automated generalistic Malaise trap with a metabarcoding workflow for species identification, we enhance our understanding of the ecology of various insect species simultaneously and enable assumptions about potential reciprocal relationships. To the best of our knowledge, this study is the first to assess circadian activity patterns from complex insect bulk samples, independent of body size and without a taxonomic emphasis on specific insect orders.

## 2 MATERIALS AND METHODS

### 2.1 Sampling

**FIGURE 1:**
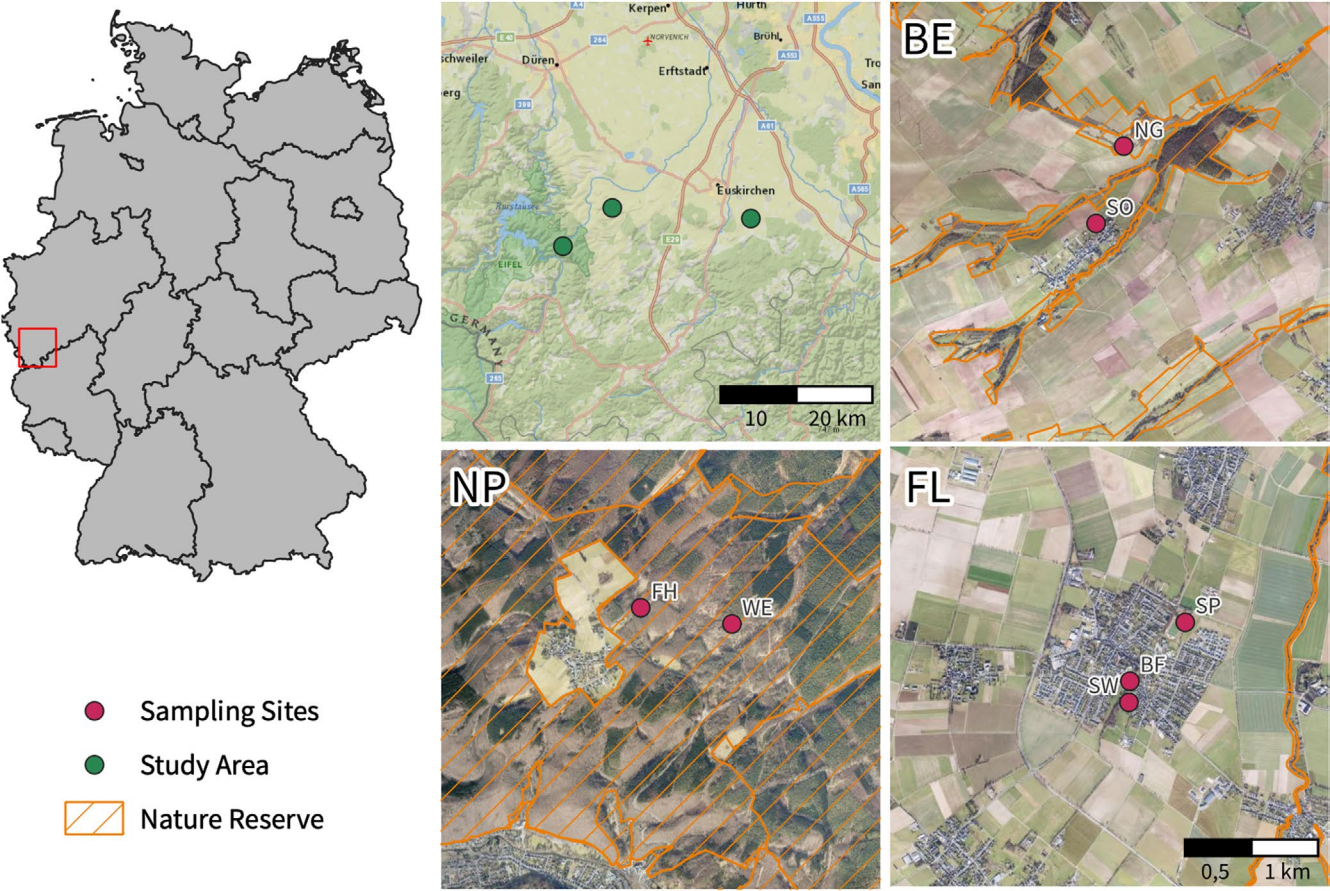
Overview of sampling locations. Sampling was conducted in the greater Eifel low mountain range, specifically in Mechernich‐Berg (BE), Euskirchen‐Flamersheim (FL) and the Eifel National Park (NP) (as indicated with green circles). The geographic location of Malaise traps is indicated with red circles. Nature reserves are marked in orange.

The study was conducted in the Eifel low mountain range in southwestern North Rhine Westphalia, Germany (Figure 1; Table 1). At the beginning of July 2022, three Malaise traps were set up in the municipalities of Euskirchen‐Flamersheim. Additionally, two Malaise traps were installed in Mechernich‐Berg and two more near the village of Schleiden‐Wolfgarten, within the Eifel National Park.

**TABLE 1:** Sampling sites.

Location	Sample site	Habitat	Latitude	Longitude
Eifel National Park	Forsthaus (FH)	Forest^a^	50.594263	6.499932
Eifel National Park	Weiher (WE)	Forest^a^	50.593173	6.509610
Mechernich‐Berg	Streuobstwiese (SO)	Orchard	50.635197	6.591573
Mechernich‐Berg	Naturschutzgebiet (NG)	Grassland^a^	50.640406	6.594506
Flamersheim	Burg (BF)	Park	50.621882	6.851964
Flamersheim	Sportplatz (SP)	Sports field	50.625846	6.857838
Flamersheim	Schafsweide (SW)	Grassland	50.620467	6.851862

*Note*: Depicted for each sampling site is the associated habitat as well as geographical coordinates (WGS84).

^a^ Nature reserve.

To monitor the circadian rhythm of insect activity, Malaise traps were equipped with an AMMOD multisampler (Kirse et al., 2024; Wägele et al., 2022). The multisampler replaces the traditional collection bottle and enables an automated time‐controlled exchange of collection bottles (Kirse et al., 2024).

For this study, the multisampler was programmed to change the collection bottles every 2 h for a total of 14 days per sampling round. Consequently, each bottle was placed under the sampling device 14 times, resulting in 12 samples per Malaise trap after 14 days. All collection bottles were filled with 500 mL of 96% denatured ethanol (1% MEK, type 641) to ensure the best possible preservation of specimens for DNA‐based species identification. Sampling season took place from 5 July 2022 until the 11 October 2022, with the first samples being collected on 19 July 2022.

Permits for insect sampling were granted from the responsible authority (Untere Naturschutzbehörde Euskirchen).

### 2.2 Laboratory work

For this study, specimen identification was conducted using an elaborated DNA metabarcoding protocol, as follows. Insect bulk samples were size‐sorted into two size fractions (S: <4 mm and L: ≥4 mm) using a stainless steel mesh (4 × 4 mm grid, wire diameter of 0.5 mm). Depending on number and size of specimens, resulting size fractions were transferred into 5‐mL tubes (Eppendorf, Hamburg, Germany), 30‐mL tubes (Nalgene, wide‐mouth bottle; Thermo Fisher Scientific, Waltham, MA, USA) or 40‐ and 100‐mL Milling tubes (IKA MT 40 and MT 100, Staufen im Breisgau, Germany), respectively. Tubes were left open and placed into an incubator (VWR, Radnor, PA, USA) at 50°C for 4–6 days allowing the ethanol to fully evaporate and the specimens to dry. Subsequently, samples were homogenised using either a Mixer mill (Retsch MM400, Haan, Germany) in combination with metal beads (3 mm for 5‐mL tubes/5 mm for Nalgene wide‐mouth bottle) for 5 min at a frequency of 30 s/s or an IKA Tube Mill 100 (Staufen im Breisgau, Germany) for 3 min at 25,000 rpm, depending on tube type used. From the resulting homogenised material, approximately 20 mg per sample were transferred into 1.5‐mL tubes (Eppendorf) for later DNA extraction.

### 2.3 DNA extraction

DNA from samples was extracted using the DNeasy 96 Blood and Tissue Kit (Qiagen, Hilden, Germany) following the manufacturer's protocol with some modifications. Briefly: 200 μL of a 5% Mastermix of ATL buffer and Proteinase K were added to each sample (S and L fraction). As negative controls, 12 reactions only including ATL and Proteinase K were processed. Samples were incubated at 56°C overnight using a shaking incubator which cycled at 110 rpm (INCU Line ILS 6; VWR, Radnor, PA, USA). The next day, 135 μL of the S fraction lysate and 15 μL of the L fraction lysate of each sample were merged and 307.5 μL of AL buffer—ethanol mixture was added to each sample. Subsequently, depending on the amount of retrievable liquid, up to 457.5 μL of the mixture was transferred into a single tube of a spin column plate, before centrifugation for 10 min at 3800 rpm. To remove any remaining contaminants and inhibitors, first 500 μL of AW1 buffer was added and spun down for 5 min at 6000 rpm and secondly 500 μL of AW2 buffer, followed by 15 min at 6000 rpm. Finally, DNA was eluted in 200 μL AE. After 1 min of incubation, samples were centrifuged for 2 min at 6000 rpm. Extraction success and DNA quality were checked on a 1% agarose gel. DNA eluate was subsequently transferred into a −20°C freezer until further processing.

### 2.4 Amplicon library preparation

A two‐step PCR protocol using the PCR Multiplex Plus Kit (Qiagen) was followed for amplicon library preparation (Kirse et al., 2023). For initial PCR, the primers fwhF2 (Vamos et al., 2017) and fol‐degen‐rev (Yu et al., 2012) were used. All PCRs were conducted with the Applied Biosystems 2720 187 thermocycler.

Initial PCR Mix (PCR 1) consisted of 12.5 μL Multiplex PCR Master Mix, 0.2 μM of the forward and 0.2 μM of the reverse primers, 10.5 μL ddH_2_O and 1 μL of DNA template. The following PCR protocol was applied: initial denaturation at 95°C for 5 min; 25 cycles of 30 s at 95, 50 and 72°C each; and a final extension period of 5 min at 72°C. For PCR 2, PCR 1 products were used as templates and Nextera tagging primer for indexing (Nextera, Illumina, San Diego, CA, USA). Again, 12.5 μL Multiplex PCR Master Mix, 0.2 μM of the forward and 0.2 μM of the reverse primers, 9.5 μL ddH_2_O and 1 μL of PCR 1 product were used for PCR, following the same PCR programme as described for initial PCR but with 15 instead of 25 cycles. PCR success was evaluated on a 1% agarose gel. PCR 2 products were normalised using a SequalPrep normalisation plate (Thermo Fisher Scientific) following the manufacturer's instruction with an end concentration of 25 ng per sample (20 μL). Subsequently, aliquots of 10 μL per sample were pooled together, before two rounds of left‐sided size selection using magnetic beads (SPRIselect; Beckman Coulter, Brea, CA, USA) and a sample to bead ratio of 1:0.7 in order to remove remaining primer remnants were undertaken. The pooled libraries were sent to Macrogen Europe (Amsterdam, The Netherlands) for sequencing on a HiSeq2500 sequencer (Illumina, San Diego, CA, USA).

### 2.5 Bioinformatics and statistical analysis

Demultiplexing was performed by Macrogen Europe. Primer pairs were eliminated using cutadapt 3.5 (Martin, 2011) with the following settings: maximum error rate (e) 0.1, minimum overlap (‐O) 20 and minimum sequence length (m) 150. Subsequently, the samples were loaded into QIIME 2 version 2023.2 (Bolyen et al., 2019). Only sequences that had both primers were kept for further analysis. Sequences were truncated to 225 bp before DADA2 (Callahan et al., 2016) was used for paired‐end read merging, quality filtering, chimaera detection and denoising. Sequences with ≥97% similarity were clustered into 8486 operational taxonomic units (OTUs) using VSEARCH (version 2.22.1) (Rognes et al., 2016). The LULU algorithm (Frøslev et al., 2017) was applied for further qualitative filtering resulting in 8186 OTUs. Taxonomic assignment was performed against the BOLD database with BOLDigger (Buchner & Leese, 2020). Result list was filtered with the JAMP‐Pipeline option.

The resulting OTU table was further analysed with the statistical software R (version 4.4.0) (R Core Team, 2013). The R packages ‘dplyr’ (Wickham et al., 2019), ‘janitor’ (Firke, 2021) and ‘reshape2’ (Wickham, 2007) were used for data wrangling. The packages ‘ggplot2’ (Wickham & Wickham, 2016), ‘cowplot’ (Wilke, 2024), ‘viridis’ (Garnier et al., 2024) and ‘RColorBrewer’ (Neuwirth & Neuwirth, 2014) were used for visualisation. Activity curves were calculated as the predicted number of detections using generalised linear latent variable models implemented in ‘gllvm’ (Niku et al., 2020) with time and sampling round as a predictor and site as a random term:

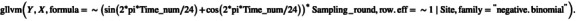


*Y* is a matrix with the number of species per taxon and sampling event, while *X* is a matrix containing the metadata for each sampling event. A binomial distribution was used for models at species level. Sine and Cosine transformations of time were implemented to account for the circular nature of time.

Similarity between communities was assessed using the Jaccard similarity index (JSI), calculated with the vegdist() function from the package ‘vegan’ (Dixon, 2003).

## 3 RESULTS

### 3.1 Influence of daytime on assessed species richness

The AMMOD multisampler is still in a developmental phase and the system has not been free of errors during its application for this study (see Table S1). In total, 430 bottles accounting for 36 complete biweekly collection trials can be used for subsequent analysis. Notably, two bottles collected at sampling site 08 (Berg Naturschutzgebiet) in sampling round 7 between 4 a.m. and 8 a.m. did not contain any insects and were therefore not metabarcoded.

**FIGURE 2:**
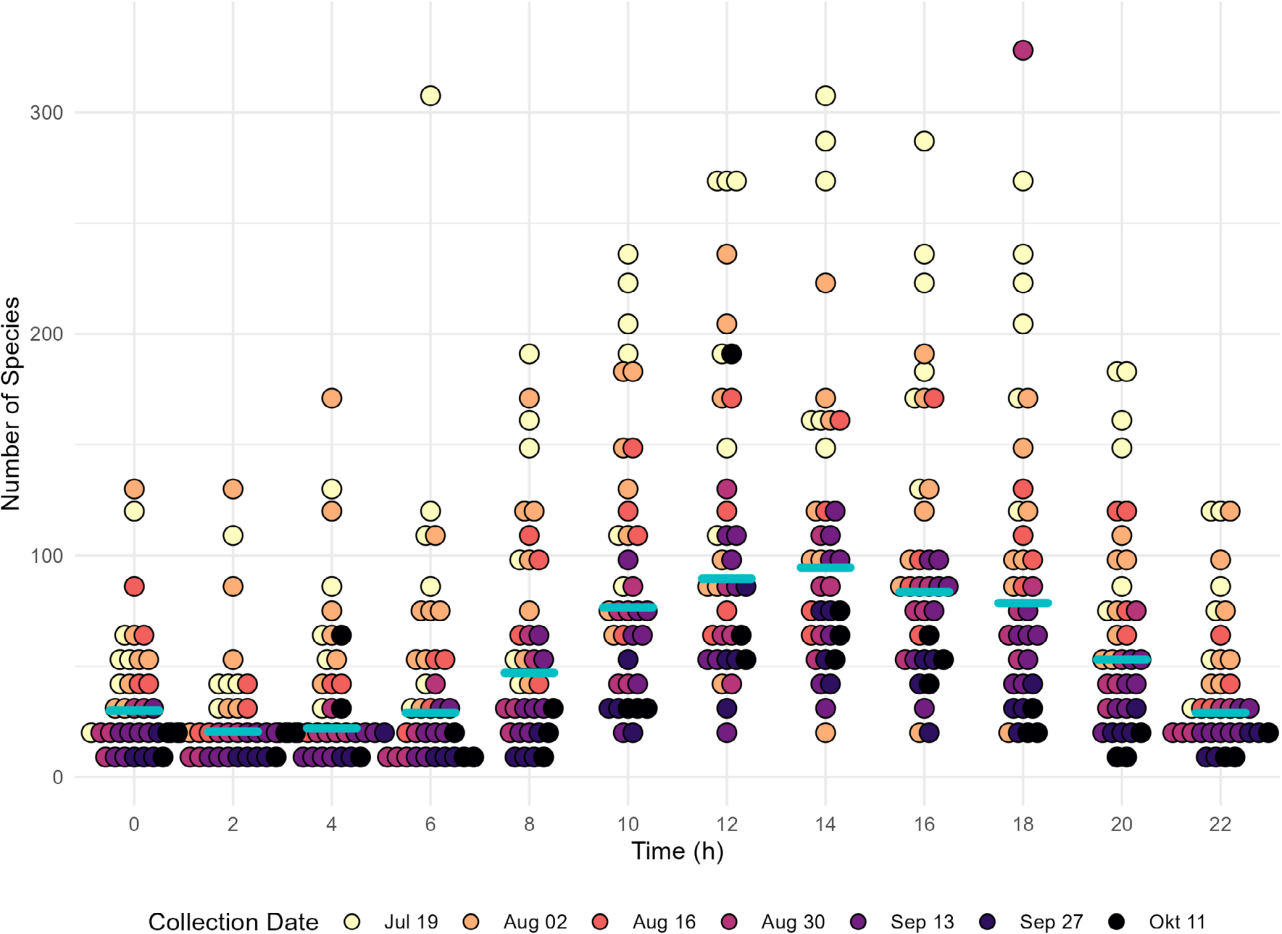
Number of insect species collected over a 24‐h sampling period for each Malaise trap and collection date (*n* = 430). Blue lines indicate the median of the number of detected species per time point. Colour shades represent the sampling rounds.

Visualisation of data suggested that the time of sampling had a pronounced impact on the number of detected species (Figure 2). On average, the lowest species count per sampling session and trap occurred between 2 a.m. and 4 a.m. Subsequently, there was a consistent rise in the number of active insect species until reaching a peak at 2 p.m. This trend was found across all sampling rounds (Figure 2).

In our dataset, each Malaise trap location underwent multiple sampling rounds, resulting in the pseudoreplication of data points within each trap. Therefore, we aggregated the data of each Malaise trap by calculating the total number of assessed species across all sampling rounds. Subsequent statistical evaluation confirmed our observation that the time of sampling had a pronounced impact on the number of identified species [Kruskal–Wallis test: *H*(11) = 143.39, *p* < 0.001].

### 3.2 Influence of daytime on taxa detection rates

**FIGURE 3:**
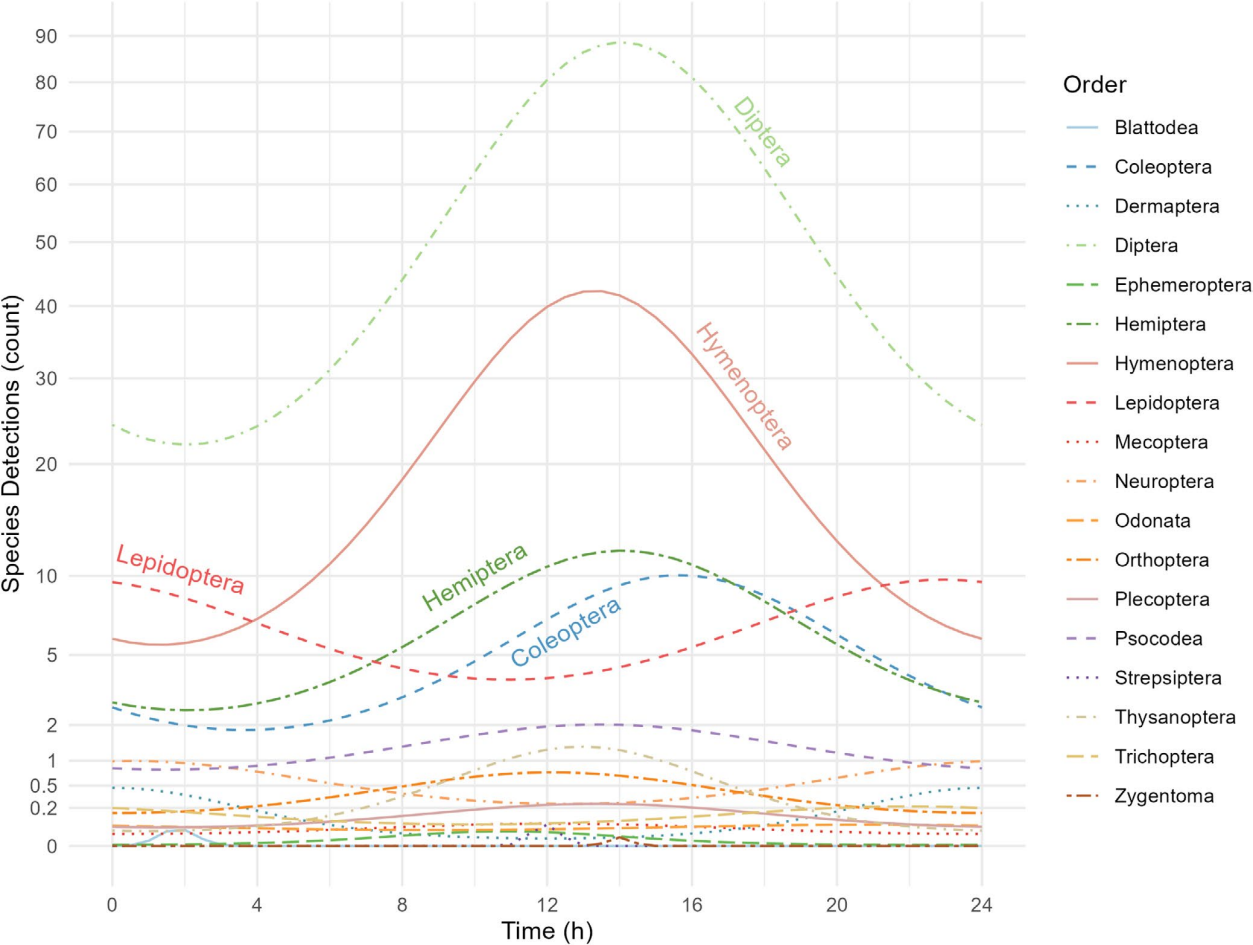
Diel activity patterns of different insect orders. Generalised linear latent variable model showing species detection patterns across insect orders throughout the day during sampling round 2 (2 August). The *y*‐axis has been square root transformed to better visualise differences among lower detection values. An alternative version with an untransformed *y*‐axis, along with plots from other sampling rounds, can be found in Figures S3 and S4.

Here, we use the number of species detections as a proxy for the activity rate of the assigned taxon. Calculated generalised linear latent variable models show that the highest number of insect species detections was found around midday between 12 p.m. and 2 p.m. (Figure 3). From here on, insect activity decreased steadily until 2 a.m. This trend was observed with slight variations for most insect orders, including Diptera, Hymenoptera, Hemiptera, Psocodea and Thysanoptera. Coleopteran activity reached a peak in the later afternoon between 2 p.m. and 6 p.m., before a very low activity was recorded at 4 a.m. In contrast, Neuropterans and Lepidopterans showed a peak in activity during night‐time, specifically between 8 p.m. and 2 a.m. (Figure 3). However, this trend differed strongly between lepidopteran families.

Overall, a total of 45 lepidopteran families were identified (see Table S2), out of which 18 families were observed on more than 20 occasions. For those 18 families, a generalised linear latent variable model was calculated to assess the expected species detection rate depending on daytime across all sites and sampling rounds.

**FIGURE 4:**
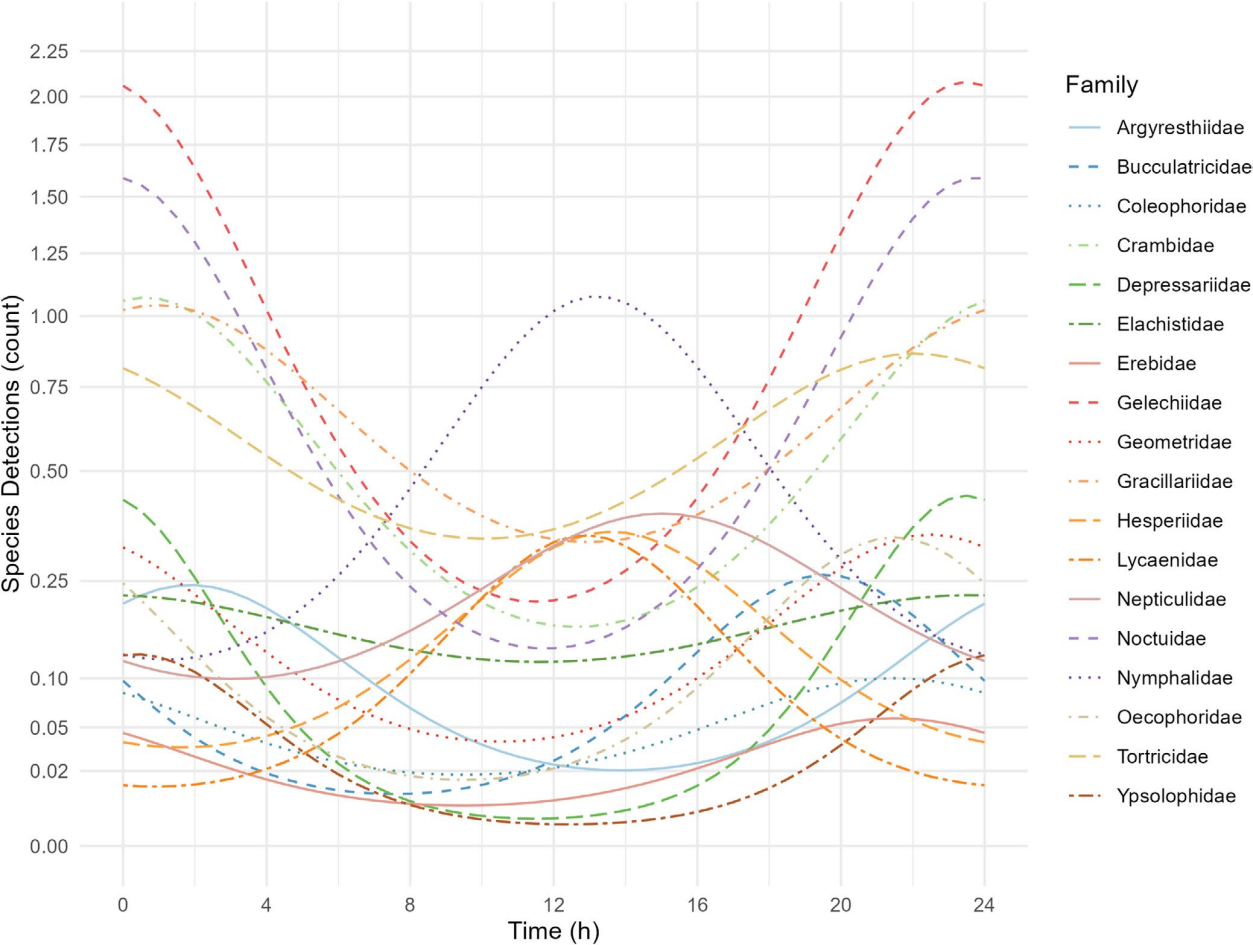
Lepidopteran diel activity patterns. Predicted detection patterns throughout the day for the 18 most frequently observed Lepidoptera families during sampling round 2 (2 August), based on a generalised linear latent variable model. Each line represents the model's predicted number of detections across different times of day for a single family. The *y*‐axis has been square root transformed to highlight differences in lower detection values. For untransformed data, see Figure S5.

The model reveals distinct temporal patterns in the detection rate of various lepidopteran families (Figure 4). Notably, species belonging to the Nymphalidae, Nepticulidae, Hesperiidae and Lycaenidae are most likely to be observed during the daytime, particularly between 12:00 p.m. and 4:00 p.m. Conversely, Bucculatricidae and Elachistidae exhibit a higher likelihood of detection in the late afternoon, specifically between 4:00 p.m. and 10:00 p.m., while Erebidae, Coleophoridae, Oecophoridae, Geometridae, Tortricidae and Depressariidae shows a peak in activity between 8 p.m. and 12 a.m. The Ypsolophidae, Argyrestiideae, Gracillaridae, Crambidae, Gelechiidae and Noctuidae show the highest detection probability during the night‐time, particularly between 10:00 p.m. and 2:00 a.m. (Figure 4).

**FIGURE 5:**
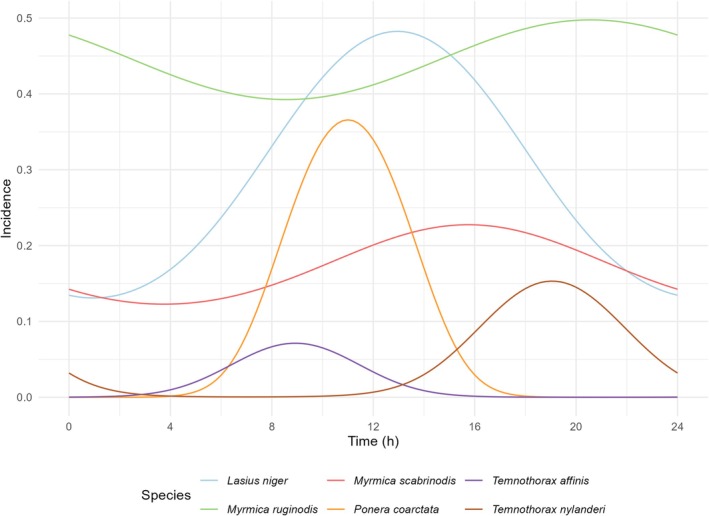
Ant activity patterns. Predicted detection numbers for six ant species (Hymenoptera: Formicidae) throughout the day during sampling round 2 (2 August), based on a generalised linear latent variable model. The species included are *Lasius niger*, *Myrmica ruginodis*, *Myrmica scabrinodis*, *Ponera coarctata*, *Temnothorax affinis* and *Temnothorax nylanderi*. For other sampling rounds, see Figure S6.

For the hymenopterans, we observed the highest predicted detection rates during daytime between 10 a.m. and 8 p.m. (see Figures S1 and S2). Noteworthy, slight temporal variations in peak activity were observed among different families. Ichneumonidae, Formicidae, Braconidae, Diapriidae, Pteromalidae, Halictidae, Proctotrupidae, Crabronidae, Pompilidae, Chrysididae, Andrenidae, Colletidae, Megachilidae, Vespidae and Tenthredinidae demonstrated heightened detection probabilities between 12 a.m. and 2 p.m. In contrast, Figitidae exhibited a peak in activity around 2 p.m., while Eulophidae reached their peak in activity at 4 p.m. Apidae Dryinidae and Pemphredonidae displayed an increased detection rate shortly before and around noon (see Figures S1 and S2). Although the highest number of detections for the Hymenoptera family Formicidae was found shortly after noon, considerable variation in activity patterns at the species level was observed within the family, where 29 ant species exhibited distinct activity profiles. While *Lasius niger*, *Myrmica ruginodis* and *Myrmica scabrinodis* displayed continuous activity throughout both day and night, others, such as *Ponera coarctata*, *Temnothorax affinis* and *Temnothorax nylanderi*, were exclusively observed in a distinct time frame (Figure 5). In detail, *Ponera coarctata* was only found between 6 a.m. and 8 p.m., *Temnothorax affinis* exhibited activity from 4 a.m. to 2 p.m. while *Temnothorax nylanderi* showed a peak in activity between 2 p.m. and 2 a.m. (Figure 5). However, it is noteworthy that *T. nylanderi* was also occasionally found in a single sample collected between 10 a.m. and 12 p.m. in one trap.

### 3.3 Influence of daylength on species richness depending on daytime

**FIGURE 6:**
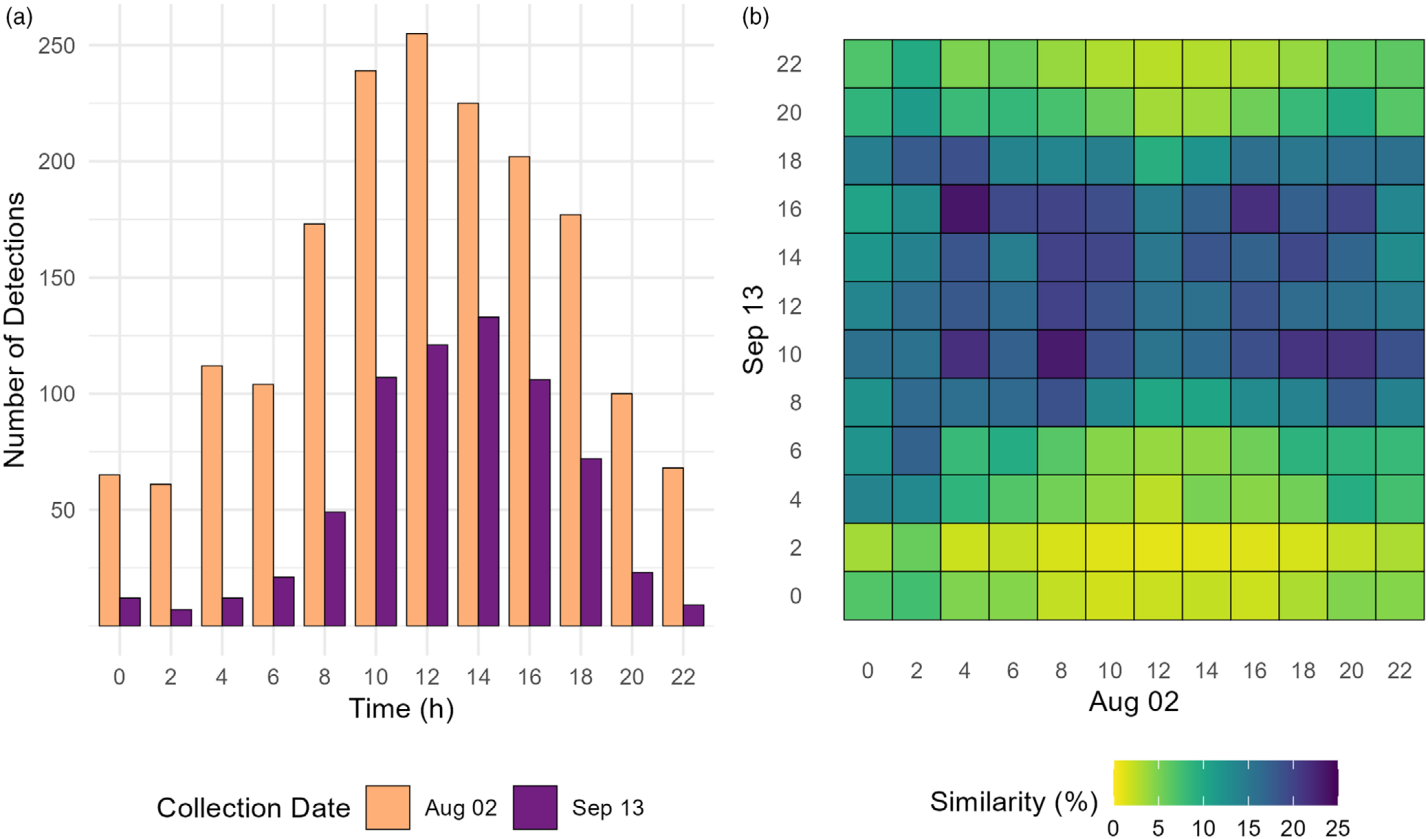
Influence of sampling round on number of Hymenoptera detections. (a) Number of Hymenoptera detections over time depicted for samples collected from 19 July until 2 August (sampling round 2) and for samples collected from 31 August until 13 September (sampling round 5); (b) heatmap showing Jaccard similarity index for hymenopteran communities collected in sampling rounds 2 and 5, respectively, depending on time. Jaccard similarity was calculated based on hymenopteran species detected in both sampling rounds.

Over the seven sampling rounds, a decline in species richness was observed (see Figure S4). In order to investigate the potential impact of variations in day length on shifts in insect activities, our focus is directed towards the diverse yet predominantly day‐active insect order Hymenoptera. A total of 631 hymenopteran species were identified during the sampling rounds 2 (19 July 2022 to 2 August 2022) and 5 (31 August 2022 to 13 September 2022). In sampling round 2, sunrise occurred at 5:07 a.m. with sunset at 8:13 p.m., whereas in sampling round 5, sunrise occurred an hour later (6:10 a.m.), and sunset approximately 90 min earlier (6:48 p.m.). Notably, in sampling round 2, a peak in hymenopteran activity was observed between 12 p.m. and 2 p.m., whereas in sampling round 5, the highest number of hymenopteran observations was recorded between 2 p.m. and 4 p.m. (Figure 6a). To assess the similarity between samples from the two sampling rounds, the JSI was calculated. Up until midday collection, the samples from sampling round 2 exhibited greater similarity to those from sampling round 5 that were collected later in the day (Figure 6b). Notably, samples taken between 8 a.m. and 10 a.m. during sampling round 2 demonstrated an overall higher similarity to samples taken between 10:00 a.m. and 12:00 a.m. in sampling round 5. From midday until 4 p.m., samples from both sampling rounds displayed the highest JSI to samples collected at the corresponding times. However, after 4 p.m., samples taken in sampling round 2 showed greater similarity to those collected earlier in the day in sampling round 5 (Figure 6b).

## 4 DISCUSSION

Automated trap systems are a powerful tool to broaden our knowledge about insect behaviour. Today, it is widely recognised that insect activity is closely tied to climate factors, such as temperature (Vicens & Bosch, 2000), humidity (Beck, 2012), rainfall (Lawson & Rands, 2019; Tuell & Isaacs, 2010), and even wind speed (Hennessy et al., 2020; Lawson & Rands, 2019). These variables can undergo significant fluctuations throughout a week, whereas it is indispensable to keep track of changes in abiotic factors to allow for conclusions of reciprocal relationships to insect activity patterns. The AMMOD multisampler model used in this study is equipped with a temperature logger which is positioned inside the sampler. Recorded values are therefore significantly affected by heat accumulation and fail to accurately reflect the air temperature in the immediate vicinity of the sampler. Consequently, this study has to rely exclusively on time‐controlled catches, yet it still offers a proof of concept for the benefits of automating sampling devices. Due to the automatisation of a generalistic flight interception trap in combination with DNA metabarcoding, this study is not limited to a small set of taxa like previous studies (Geissmann et al., 2022; Lamarre et al., 2015; Zoller et al., 2020), whereas the produced dataset can provide valuable information about species ecology and allows for assumptions on possible reciprocal relationships.

Consistent with prior research, we have observed that insect activity follows circadian rhythms (Knop et al., 2018; Saunders, 2002; Strumia, 2011). In their work dating back to 1965, Lewis and Taylor investigated activity patterns in approximately 400 insect taxa. They discovered similar patterns, where Coleoptera, Hymenoptera, Thysanoptera and Hemiptera exhibited heightened activity during daylight hours, while Lepidopterans, Trichopterans and Neuropterans were predominantly active during the night.

Previous research has demonstrated variations in lepidopteran diel activity patterns across families (Lamarre et al., 2015). The dataset presented in this study provides a comprehensive examination of diel activity pattern variances at both the family and species levels. Here, a total of five species of the lepidopteran family Hesperiidae were detected, namely *Ochlodes sylvanus*, *Spialia sertorius*, *Thymelicus acteon*, *Thymelicus lineola* and *Thymelicus sylvestris*. All of them are described to be exclusively active during daylight hours. However, *Thymelicus sylvestris* was found in five samples collected between dusk and dawn (8 p.m.–4 a.m.) at a single location (MT02) over two consecutive sampling rounds (1 and 2). A previous study has noted occasional nocturnal activity of *T. lineola*, a species closely related to *T. sylvestris*. However, the authors describe those observations as exceptionally rare (Fullard & Napoleone, 2001). Remarkably, in our study, only at MT02 nocturnal activity was observed for *T. sylvestris*. This finding may suggest either a unique expression of nocturnal activity within this population or a higher frequency of disturbances during night‐time. MT02, situated in a small forest clearing covered with tall grass, is recognised for the regular grazing and resting behaviours of both red deer and roe deer, providing support for the latter assumption.

In contrast to the Hesperiidae the Oecophoridae are described as mostly nocturnal or crepuscular (Harper et al., 2002). Here, in total, eight species were found. Out of those, only the species *Borkhausenia fuscescens* was observed between 6 a.m. and 4 p.m. Both observations occurred in sampling round 3 but at two different locations (MT08 and MT12). This suggests that the *B. fuscescens* shows occasional activity patterns throughout daylight. Such diurnal activity patterns have also been described for other Oecophoridae species (Harper et al., 2002; Peterson et al., 2007), but we could not find any publication dealing with the activity pattern of this specific species under field conditions. Overall, for most moth species, little is known about behaviour and ecology, which is likely also attributed to the high workload and the associated elevated financial expenditures which come with associated research.

Some observations may be influenced by cross‐contamination, potentially due to regurgitation from predatory insect species. It is well known that certain insects tend to regurgitate when captured in ethanol‐filled trap systems (Kirse et al., 2023). However, in this study, a destructive DNA extraction protocol was applied, producing a DNA extract in which the quantity of community DNA (cDNA) directly released from the grinded specimens markedly exceeded the environmental DNA (eDNA) in the sample (Iwaszkiewicz‐Eggebrecht et al., 2022; Kirse et al., 2023). This effect was further enhanced by washing all specimens with fresh ethanol during sieving, which likely removed additional eDNA residues from surfaces of specimens.

Next to a wide variety of lepidopteran species, we have identified a total of 951 hymenopteran species (see Table S2) belonging to 56 families. In comparison with previous comparable metabarcoding studies based on bulk samples collected with Malaise traps, the number of identified hymenopteran species is exceptionally high (Kirse et al., 2023; Zizka et al., 2022). Several studies have already shown that hymenopterans are one of the most challenging groups to target with metabarcoding, inter alia due to inconsistencies in degree of conservation in the primer binding region (Brandon‐Mong et al., 2015; Elbrecht et al., 2019; Kirse et al., 2021). Here, the impact of primer bias was likely reduced through the presorting of samples. Unlike traditional Malaise trap samples, which exhibit higher diversity, in this case, insects were sorted into distinct bottles based on their activity patterns. As a result, the proportion of hymenopterans in bottles collected during midday increased as well as the linked number of hymenopteran sequences in bulk DNA, directly impacting detection probability. Overall, increasing the detection rate of hymenopterans when metabarcoding bulk samples is desirable.

Many hymenopterans, including several members of the family Ichneumonidae, belong to the so‐called ‘Dark Taxa’, which are taxa that are not or scientifically inadequately documented whereas little is known about their role and benefits in natural and anthropogenic ecosystems, as well as their threat status (Rduch & Peters, 2020). Here, a total of 404 Ichneumonidae species were identified. Out of those 28 were observed between 12 p.m. and 2 a.m. and 32 between 2 a.m. and 4 a.m., respectively. The species *Stenomacrus affinitor* was found at both time periods in two Malaise trap samples, while the remaining four *Stenomacus* species were exclusively observed during daylight hours (4 a.m.–10 p.m.). However, the number of observations of *S. affinitor* increased until midday when a peak was reached with a total of 11 observations between 12 a.m. and 2 p.m. Observations could either point to a more generalistic host choice or to a specialisation on a host species with similar activity rhythms. *Stenomacrus* species are known as koinobiont endoparasitoids of dipterans and especially of fungus gnats (Sciaridae) (Woelke et al., 2020). However, in the scientific literature, little can be found of verified host–parasitoid relationships. Here, a total of 102 Sciaridae species were identified, with only 19 and 18 species between 12 p.m. and 2 a.m. and 2 a.m. and 4 a.m., respectively. Among these, 12 species were found at both time points, six of which belonged to the Sciaridae genus *Bradysia*. Notably, only *B. trivitatta* was observed in sampling round 4, when *S. affinitor* was found in the same location (National Park Eifel), albeit not in the same trap. Furthermore, both potential host and associated parasitoids were detected during day‐ and night hours, suggesting a potential host–parasitoid relationship between the two species. This hypothesis is reinforced by a recent study demonstrating that the genus *Bradysia* serves as a host for certain *Stenomacrus* species (Woelke et al., 2020). However, it is important to note that the automated Malaise trap sampler used in this study only allows for the observation of the imagines of *B. trivitatta*, while *S. affinitor* attacks the larvae. The activity patterns of the larval stage of *B. trivitatta* remain poorly understood. Laboratory experiments have indicated that the activity of *Bradysia* larvae is primarily driven by hunger. Parasitoids typically utilise host‐related chemical cues to detect potential hosts (Dicke et al., 1990), and these volatiles are commonly released by plants under attack by herbivorous larvae (Dickens, 1999; Kessler & Baldwin, 2001). This suggests a direct relationship between feeding activity, volatile release and parasitoid activity. Here, the observed co‐occurrence of parasitoids and Imagines of the potential host species could indicate that activity patterns of larvae and Imagines of *B. trivitatta* are linked. However, to comprehensively understand these relationships additional field research focusing on the activity patterns of herbivorous larvae is needed.

Next to the analysis of potential host–parasitoid relationships, automated time‐controlled sampling allows for insights into species ecology. The hymenopteran family Formicidae accounted for 29 species. Ants, like other Hymenoptera, exhibit eusocial behaviour, forming complex colonies often through nuptial flights for mating (Noordijk et al., 2008). These flights optimise outbreeding, nest selection and colony establishment (Kaspari et al., 2001). Thus, the synchronisation of these flights within ant colonies is a critical factor, and therefore often characterised by high precision and constancy (Andersen, 1991; Feitosa & Aguiar, 2016). In a broad summary, nuptial flights fall into two categories: (1) ‘male aggregation’ where males gather in one location waiting for and attracting females and (2) ‘female calling’ where females actively attract males with pheromones (Noordijk et al., 2008).

The flying forms of species using female calling for reproduction are only active for a comparatively short period of time, until the pheromone‐emitting female has mated. Afterwards, no more pheromones are released, which leads to a decline in flight activity at the respective site. In contrast, males of species following a male aggregation strategy will not move away from the aggregation sites after mating (Hölldobler & Wilson, 1990). Both behaviors are reflected in the data collected here. While three ant species known to follow the female calling strategy were only found for a comparatively short period of time, the three ant species that use male aggregation as a reproductive strategy were found regardless of the time of day, indicating that the males remain at the mating sites for a longer period of time and wait for the females to pass by.

In detail, it was observed that *Myrmica ruginodis* was detected in samples of each time period; however, initial visual analysis indicated high accumulation rates in specific samples. This pattern was sustained by calculated RRA, which showed that *M. ruginodis* accounts for up to 80% of reads in highly diverse samples between 4 p.m. and 12 a.m. A high RRA indicates an accumulation of individuals in the samples, pointing to male aggregation, which has already been described for this species (Noordijk et al., 2008). Similar but less pronounced observations were made for the species *Lasius niger* and *Myrmica scabrinodis*, for which male aggregation is also well known (Noordijk et al., 2008).

In contrast, we found that *Temnothorax nylanderi* was mainly caught between 6 p.m. and 12 a.m. However, the number of reads did not account for more than 2.5% of reads per sample on any occasion, indicating a less pronounced accumulation of individuals per sample pointing to female calling. *Temnothorax affinis* activity was recorded between the early morning hours and midday (4 a.m.–2 p.m.), indicating a differing preferred time for nuptial flight. With a maximum of 1.9% of the total read count no extensive peaks in the number of reads were observed, again pointing to female calling, a behaviour which has already been described in previous studies (Noordijk et al., 2008).

Because of recurrent failures in the AMMOD multisampler, uninterrupted monitoring data for all locations are lacking, making it impossible to conduct a thorough analysis of potential shifts in insect daily activity over the entire sampling period. The failures resulted from a deficient energy supply, particularly during prolonged adverse weather conditions. As a consequence, the batteries were only partially recharged, leading to an insufficient energy supply that could not sustain the system overnight. This issue was particularly noticeable in the first and second‐generation of the multisampler, where the built‐in batteries, stored inadequately over the cold winter months, were already several years old. Consequently, the charging capacity of these batteries had significantly deteriorated, preventing them from reaching a full charge even under optimal weather conditions. By replacing these batteries and making slight adjustments to the code, including activating the sleep mode for various modules, the inadequate energy supply issue in the multisampler has now been resolved.

Only in sampling rounds 2 and 5, none of the samplers failed, limiting analysis of potential shifts in activity patterns to those two events. Hymenoptera species present in both sampling rounds revealed a shift and overall activity within a shorter time frame. Several studies have previously indicated that insect activity is predominantly influenced by two abiotic factors: light (Saunders, 2022) and temperature (Taylore, 1963).

Historical weather data, obtained from weather stations in Nideggen (for temperature) and Heimbach (for rainfall) both situated in the greater Eifel region, were utilised to evaluate weather conditions during sampling periods. In sampling round 2, temperatures exceeding 30°C were recorded on 2 days, whereas in sampling round 5 no exceptionally warm temperatures were documented (>30°C). Throughout both sampling rounds, the temperature consistently stayed above 10°C. The mean temperature in the broader Eifel region was approximately ~20.4°C during sampling round 2 and ~18.4°C during sampling round 5, but a conducted *t*‐test indicates no significant variation between the two sampling events. In addition to temperature, rainfall can significantly influence insect activity (Poulsen, 1996). Sampling round 2 experienced rain on 6 days, while sampling round 5 had rain on 7 days. Nevertheless, with an average daily rainfall of approximately 1.67 and 1.75 L/m^2^ in the respective rounds, no significant difference were observed.

Nevertheless, it is important to note that weather data were not individually assessed at each sampling site. Consequently, local variations cannot be considered, introducing a significant degree of uncertainty. Nonetheless, relying on the provided data, the number of daylight hours appears to be the primary factor contributing to the observed shift in species activity patterns. This observation is in alignment with several other studies showing that the number of daylight hours significantly affects and moreover seems to be the main predictor for insect activity. This is further supported by the current observation that climate change distorts the alignment of flowering times and pollinator flight activity (Freimuth et al., 2022). Plants show a higher degree of sensitivity to changes in temperature leading to earlier flowering times (Freimuth et al., 2022) while, depending on taxon, pollinators potentially also utilise daylight hours as a predictor for the upcoming season.

## 5 CONCLUSIONS

In this study, it is demonstrated that the use of an automated, time‐controlled trap system in combination with metabarcoding allows a deep and previously often impossible insight into insect activity patterns. By capturing circadian rhythms of insect activity from bulk samples, it is possible to increase our knowledge about species ecology and temporal interactions, such as potential host–parasitoid interactions. This contributes significantly to the development of the field of chronoecology and thereby allowing to further investigate the role and benefits of certain species in natural and anthropogenic ecosystems, as well as their potential threat which is of tremendous importance, especially in light of the current dramatic decline in insect populations.

## AUTHOR CONTRIBUTIONS

A. Kirse, N. W. Noll and W. J. Wägele conceived the original idea for the experiment. A. Kirse and M. A. Wittenhorst planned the study. A. Kirse, M. A. Wittenhorst and D. Ott carried out the field work. M. A. Wittenhorst, A. Scherges, M. Posanski and A. Kirse carried out the laboratory work. A. Kirse, M. A. Wittenhorst, C. Scherber and V. Zizka contributed to the interpretation of the results. A. Kirse took the lead in writing the manuscript. All authors provided critical feedback and helped shape the research, analysis and manuscript.

## FUNDING INFORMATION

This work is an outcome of the AMMOD project funded by the German Federal Ministry of Education and Research (grant number 01LC1903A).

## CONFLICT OF INTEREST STATEMENT

All authors declare no competing interests.

## Supporting information


**Figure S1.** Diel activity patterns of the 20 most frequently observed hymenopteran families for sampling round 2 (August 2nd), based on a generalised linear latent variable model. Note the square root transformed y‐axis to highlight patterns in famili.
**Figure S2.** Diel activity patterns of the 20 most frequently observed hymenopteran families over seven consecutive 2‐week sampling rounds.
**Figure S3.** Diel activity pattern s of different insect orders.
**Figure S4.** Diel activity patterns across insect orders over seven consecutive sampling rounds.
**Figure S5.** Diel activity patterns for the 18 most frequently observed lepidopteran families over seven consecutive 2‐week sampling rounds, based on a generalized linear latent variable model.
**Figure S6.** Diel activity patterns day for six ant species (Hymenoptera: Formicidae) over seven consecutive 2‐week sampling rounds, based on a generalized linear latent variable model.
**Table S1.** Sampling sites and dates. Indicated with an ‘x’ are dates (columns) on which samples were successfully collected at the associated sample site (rows).
**Table S2.** OTU table.

## Data Availability

Raw sequence data for this project are available in the NCBI's SRA archive under accession number: PRJNA1119978.
