# Supplementary material for: The clockwork of insect activity: Advancing ecological understanding through automation
Source: J Anim Ecol. 2025 Jan 24;94(4):597–610. doi: 10.1111/1365-2656.14246 (PMC11962232; doi:10.1111/1365-2656.14246)
Supplement: Supplementary file 1 — Figure S1. Diel activity patterns of the 20 most frequently observed hymenopteran families for sampling round 2 (August 2nd), based on a generalised linear latent variable model. Note the square root transformed y‐axis to highlight patterns in famili. Figure S2. Diel activity patterns of the 20 most frequently observed hymenopteran families over seven consecutive 2‐week sampling rounds. Figure S3. Diel activity pattern s of different insect orders. Figure S4. Diel activity patterns across insect orders over seven consecutive sampling rounds. Figure S5. Diel activity patterns for the 18 most frequently observed lepidopteran families over seven consecutive 2‐week sampling rounds, based on a generalized linear latent variable model. Figure S6. Diel activity patterns day for six ant species (Hymenoptera: Formicidae) over seven consecutive 2‐week sampling rounds, based on a generalized linear latent variable model. Table S1. Sampling sites and dates. Indicated with an ‘x’ are dates (columns) on which samples were successfully collected at the associated sample site (rows). Table S2. OTU table. [file JANE-94-597-s001.zip › jane14246-sup-0001-Supinfo.docx]

Supplementary

# **The Clockwork of Insect Activity: Advancing Ecological Understanding through Automation**


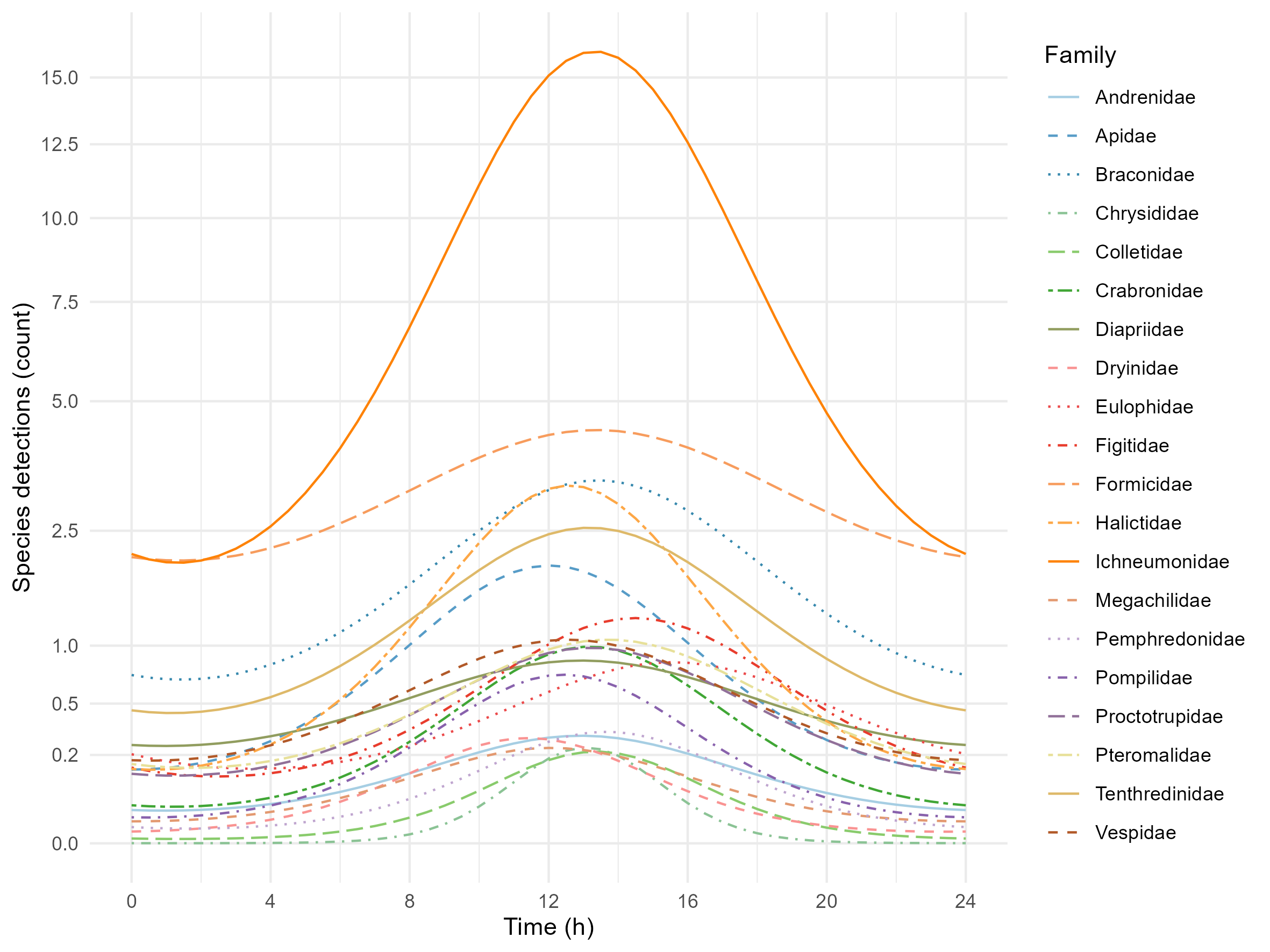


Supplementary Figure S1 - Diel activity patterns of the 20 most frequently observed Hymenopteran families for sampling round 2 ( August 2^nd^), based on a generalized linear latent variable model. Note the square root-transformed y-axis to highlight patterns in families with lower detection rates


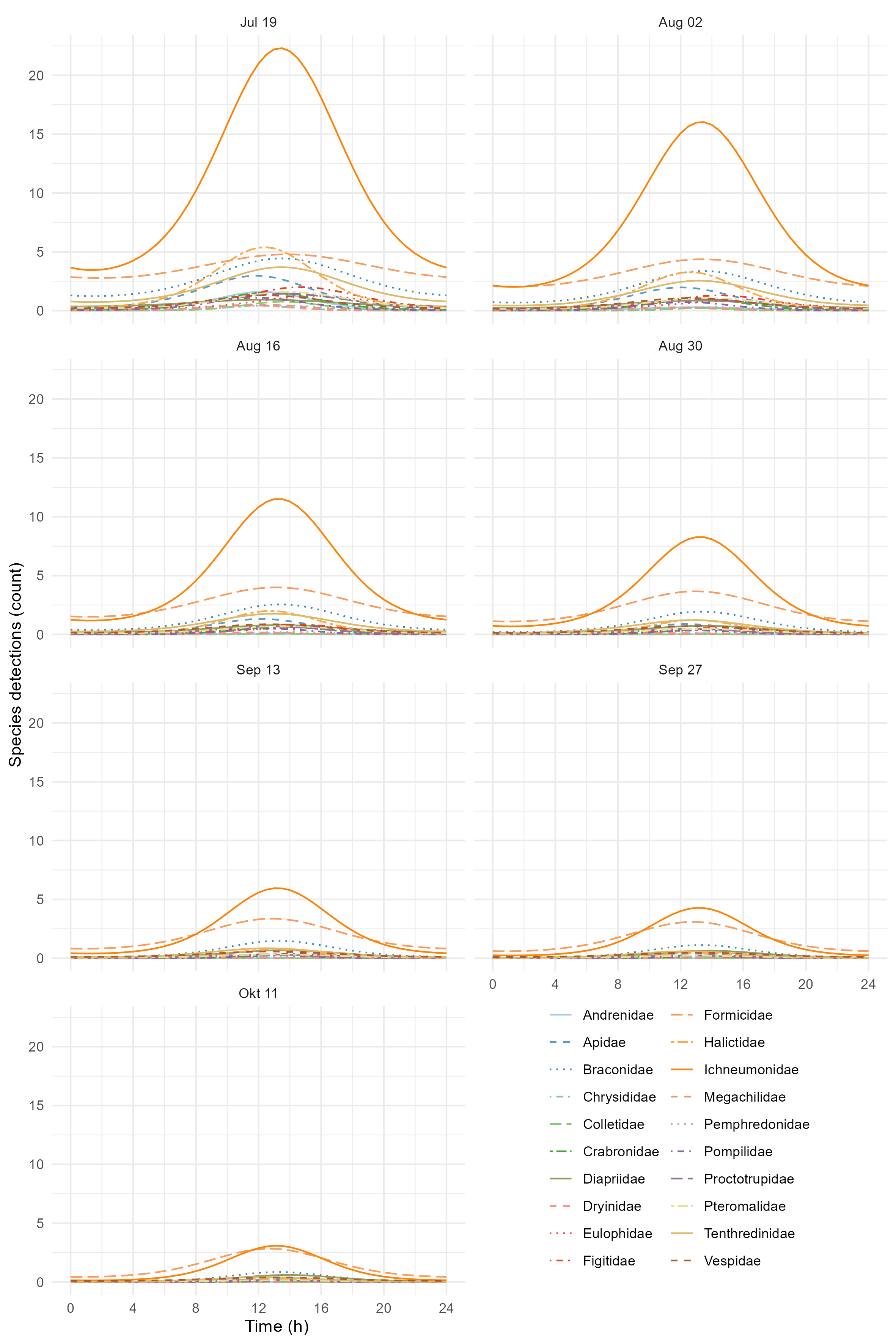


Supplementary Figure S2 - Diel activity patterns of the 20 most frequently observed Hymenopteran families over seven consecutive two-week sampling rounds. Each panel represents a distinct sampling period and shows the predicted detection patterns throughout the day for different Hymenopteran families, based on a generalized linear latent variable model.


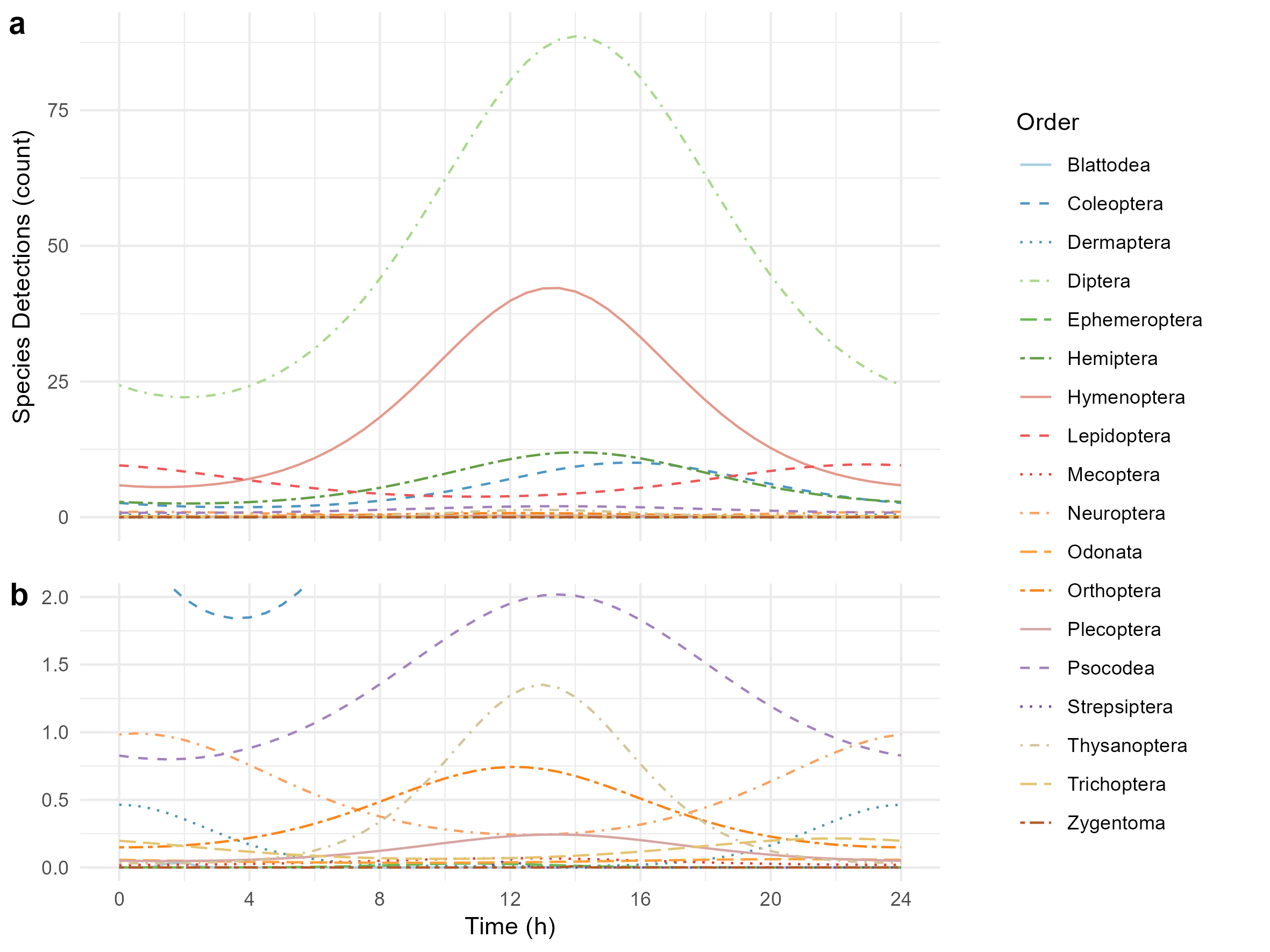


Supplementary Figure S3 - *Diel activity patterns of different insect orders*. Diel activity patterns across insect orders during sampling round 2 (August 2nd), based on a generalized linear latent variable model. Analogous to Figure 3 but with (a) showing detection patterns for all insect orders and (b) showing a detailed view of detection patterns for less frequently observed orders, using a zoomed in scale.


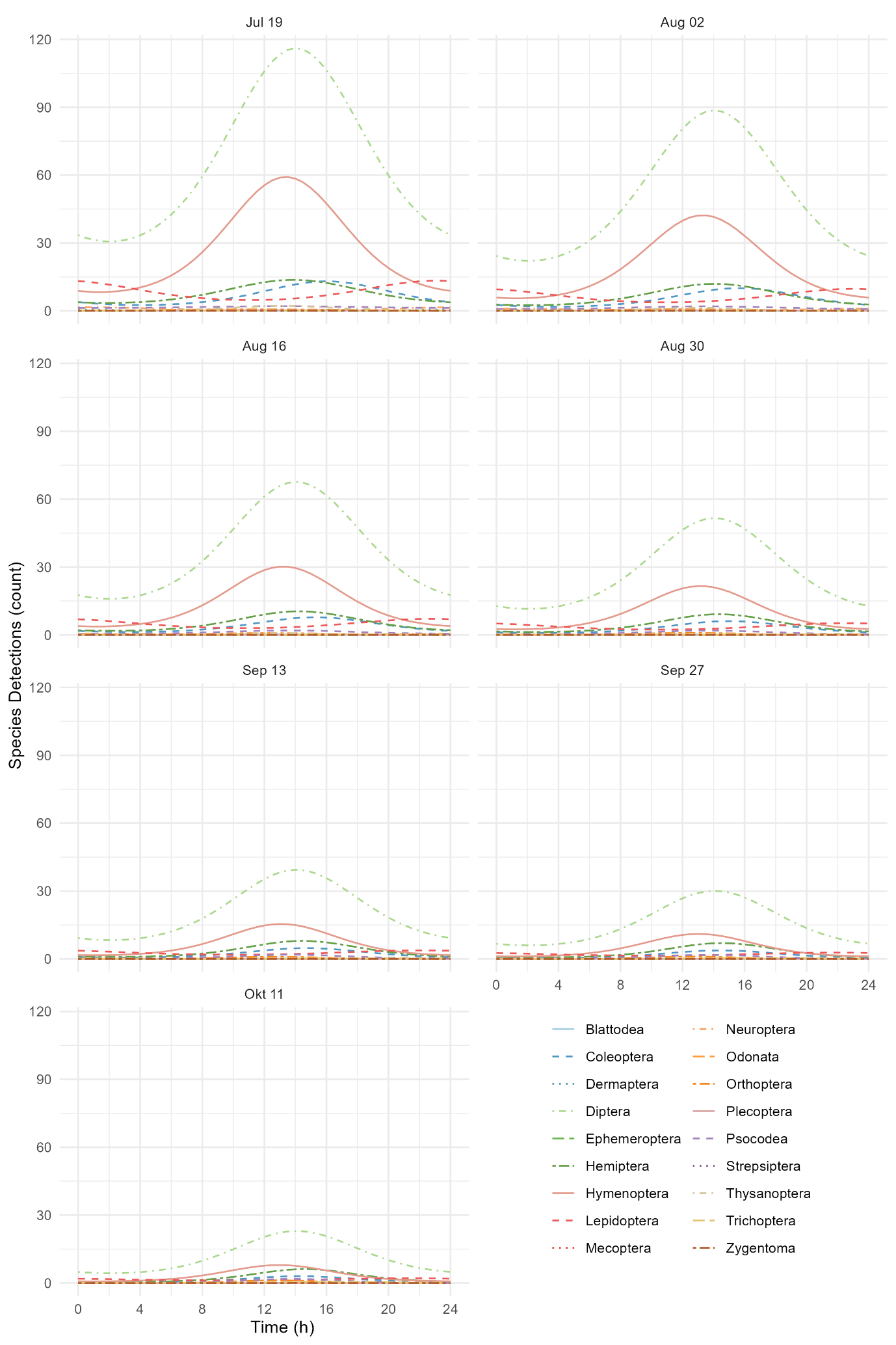
Supplementary Figure S4 - *Diel activity patterns across insect orders over seven consecutive two-week sampling rounds*. Each panel represents a distinct sampling period and shows the predicted species detection patterns throughout the day for different insect orders, based on a generalized linear latent variable model.


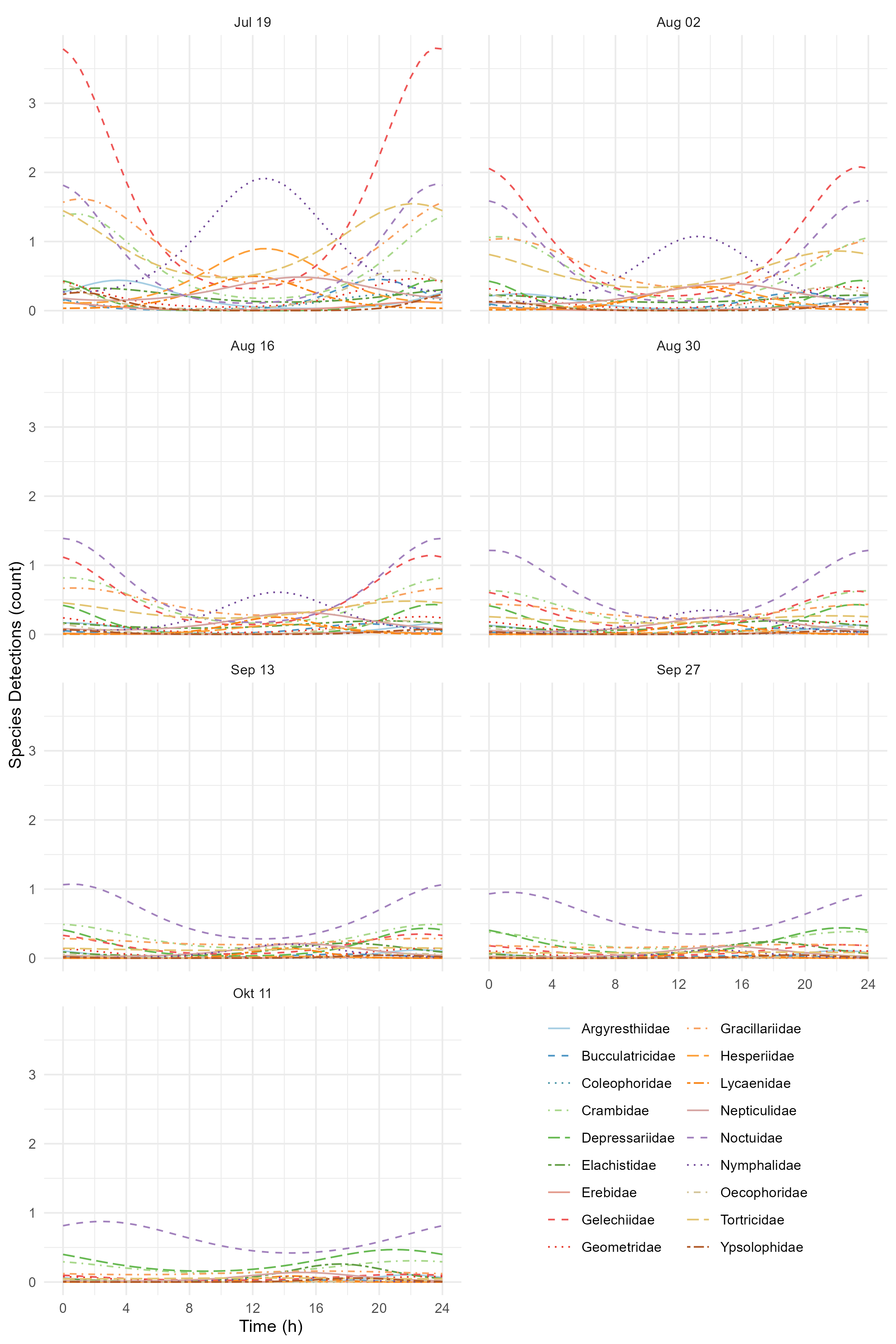


Supplementary Figure S5 - Diel activity patterns for the 18 most frequently observed Lepidoptera families over seven consecutive two-week sampling rounds, based on a generalized linear latent variable model. Each panel represents a distinct sampling period. Panel ‘Aug 02’ is analogous to Figure 4 but with an untransformed linear y-axis.


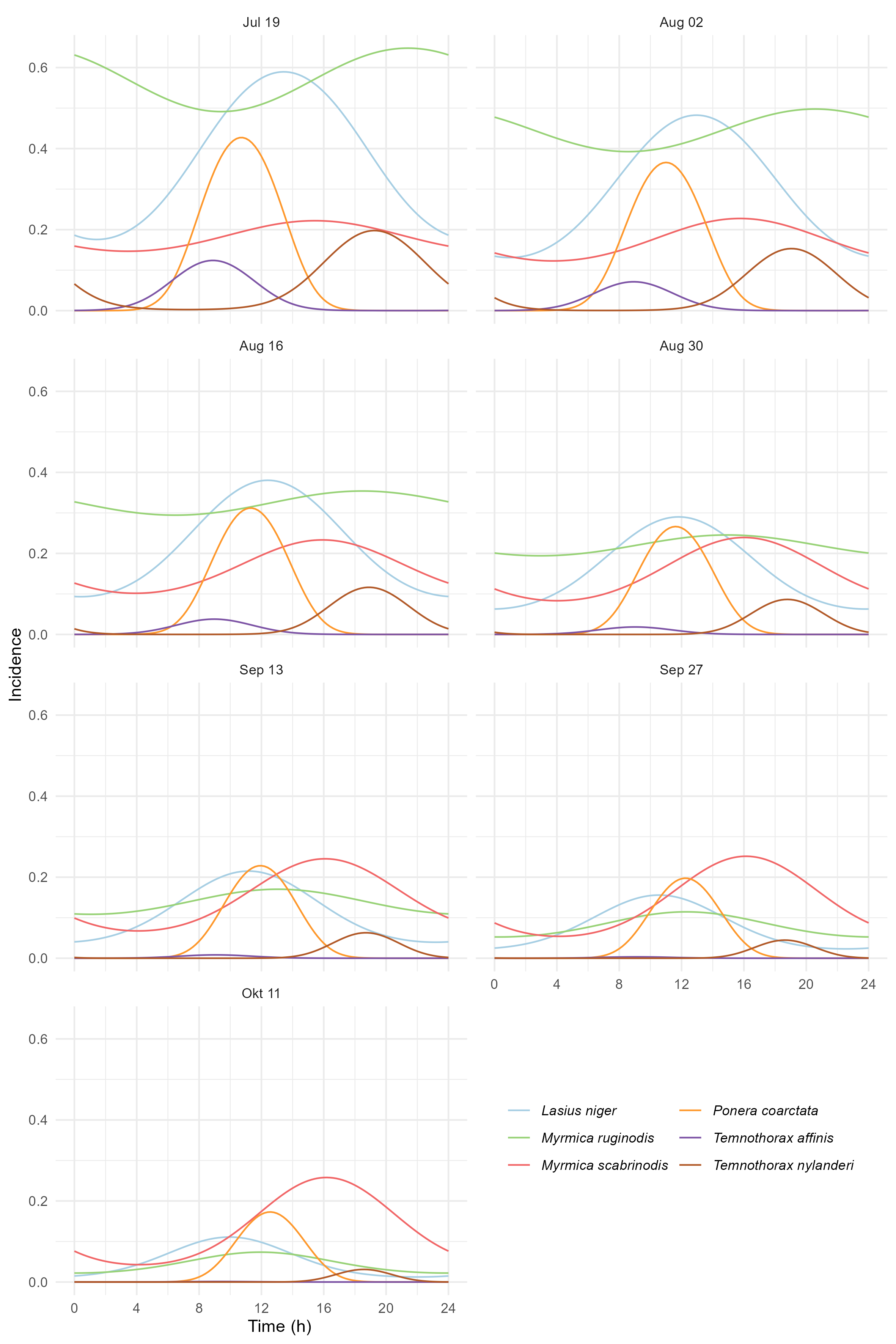


Supplementary Figure S6 - Diel activity patterns day for six ant species (Hymenoptera: Formicidae) over seven consecutive two-week sampling rounds, based on a generalized linear latent variable model. Each panel represents a distinct sampling period. Panel ‘Aug 02’ is analogous to Figure 5.

Supplementary Table S1 - Sampling sites and dates. Indicated with an ‘x’ are dates (columns) on which samples were successfully collected at the associated sample site (rows).

| Sample Site | 19.07.22 | 02.08.22 | 16.08.22 | 30.08.22 | 13.09.22 | 27.09.22 | 11.10.22 |
| --- | --- | --- | --- | --- | --- | --- | --- |
| Forsthaus | x | x | x | x | x | x | x |
| Weiher | x | x |  | x | x | x | x |
| Streuobstwiese | x | x |  | x | x |  |  |
| Naturschutzgebiet | x | x | x | x | x | x | x |
| Burg | x | x | x | x | x |  |  |
| Sportplatz | x | x | x |  | x | x |  |
| Schafsweide |  | x |  |  | x |  |  |

Supplementary Table S2 - OTU Table
